# MaAzaR Influences Virulence of *Metarhizium acridum* against *Locusta migratoria manilensis* by Affecting Cuticle Penetration

**DOI:** 10.3390/jof10080564

**Published:** 2024-08-09

**Authors:** Geng Hong, Siqing Wang, Yuxian Xia, Guoxiong Peng

**Affiliations:** 1 Genetic Engineering Research Center, School of Life Sciences, Chongqing University, Chongqing 401331, China; 17602379203@163.com (G.H.); 1330878355@163.com (S.W.); yuxianxia@cqu.edu.cn (Y.X.); 2 Chongqing Engineering Research Center for Fungal Insecticide, Chongqing 401331, China; 3 Key Laboratory of Gene Function and Regulation Technologies under Chongqing Municipal Education Commission, Chongqing 401331, China

**Keywords:** Zn(II)2Cys6 transcription factor, *Metarhizium acridum*, virulence, cuticle penetration, RNA-seq

## Abstract

The entomopathogenic fungus (EPF) *Metarhizium acridum* is a typical filamentous fungus and has been used to control migratory locusts (*Locusta migratoria manilensis*). This study examines the impact of the Zn(II)2Cys6 transcription factor, MaAzaR, in the virulence of *M. acridum*. Disruption of *MaAzaR* (Δ*MaAzaR*) diminished the fungus’s ability to penetrate the insect cuticle, thereby decreasing its virulence. The median lethal time (LT_50_) for the Δ*MaAzaR* strain increased by approximately 1.5 d compared to the wild-type (WT) strain when topically inoculated, simulating natural infection conditions. Δ*MaAzaR* compromises the formation, turgor pressure, and secretion of extracellular hydrolytic enzymes in appressoria. However, the growth ability of Δ*MaAzaR* within the hemolymph is not impaired; in fact, it grows better than the WT strain. Moreover, RNA-sequencing (RNA-Seq) analysis of Δ*MaAzaR* and WT strains grown for 20 h on locust hindwings revealed 87 upregulated and 37 downregulated differentially expressed genes (DEGs) in the mutant strain. Pathogen–host interaction database (PHI) analysis showed that about 40% of the total DEGs were associated with virulence, suggesting that MaAzaR is a crucial transcription factor that directly regulates the expression of downstream genes. This study identifies a new transcription factor involved in EPF cuticle penetration, providing theoretical support and genetic resources for the developing highly virulent strains.

## 1. Introduction

Pests like locusts, grasshoppers, termites, and cattle ticks have caused enormous economic and agricultural losses in the world [1,2,3,4,5,6,7]. In order to control these pests and vectors, chemical insecticides are frequently used as a solution [8]. However, the application of chemical insecticides has resulted in high environment pollution and negative effects on natural predators of pests [5,9,10,11,12,13]. In addition, the development of resistance in these pests has prompted in-depth research into replacing chemical insecticides with biological control agents such as fungi, bacteria, viruses, and nematodes [5,7,9,14,15,16,17,18]. Entomopathogenic fungi (EPF) infect insects by direct contact of the cuticle, unlike bacteria or viruses, which have to be ingested by an insect [19,20]. EPF do not cause environmental pollution and can even remove toxic contaminants such as alkylphenols, organotin compounds, pesticides, and hydrocarbons [19], so they are viewed as a viable alternative to chemical insecticides. Among them, *Metarhizium* spp. and *Beauveria* spp. are the most studied and widely used insecticidal fungi.

*Metarhizium acridum* has been widely studied world-wide as a model for studying insect–fungal interactions and as a genetic resource for biotechnology [21]. The key stages in the invasive and developmental processes of EPF are as follows: spore adhesion, germination, differentiation of appressoria, penetration, colonization of the hemocoel, and sporulation on insect cadavers [22]. *M. acridum* has been successfully used to control locust populations in Northern China. From 2002 to 2006, ground and aerial applications covered 6000 hectares of grasslands and the adjacent beach along the Yellow River, killing over 90% of *Locusta migratoria manilensis* (Meyen) within 11–15 days post-treatment [23]. The host’s immune response to EPF results in a long period of EPF pathogenicity, limiting its practical application. During penetration, appressoria exert significant physical force and secrete proteases like subtilisin-like proteases (Pr1) and trypsin-like proteases (Pr2 and Try1) to hydrolyze the host cuticle [24]. Insects engage in cuticular sclerotization by converting polyphenol and quinone into melanin through the action of phenoloxidases (POs). In addition, POs directly attach to and melanize fungal cell walls, thereby impeding mycelial growth [25]. Once EPF invade the hemolymph, they multiply as hyphal bodies (HBs). Hosts defend against HBs via both humoral response and cellular response, including antimicrobial peptides (AMPs), phagocytosis, encapsulation, and so on. EPF produce the collagen-like protein Mcl1, which encapsulates the surface of HBs to evade the host’s immune response [26]. Therefore, it is important to understand the mechanisms behind EPF infection of insects.

The Zn(II)2Cys6 binuclear cluster domain (IPR001138, PF00172) is unique to the fungal kingdom [27]. This multifunctional factor regulates numerous cellular processes, including carbon and nitrogen utilization [28,29], amino acid metabolism [27], and pyrimidine metabolism [30]. It also influences virulence in a lot of pathogenic fungi. UvZnFTF1 is involved in *Ustilaginoidea virens* vegetative growth, conidiation, pigment biosynthesis, and pathogenicity [31]. Tpc1 is an important Zn(II)2Cys6 transcriptional regulator necessary for polarized growth and virulence in the rice blast fungus *Magnaporthe oryzae* [32]. BbTpc1 in *Beauveria bassiana*, the homolog gene of Tpc1 in *M. oryzae*, is involved in chitin biosynthesis, fungal development, and virulence [33]. Azaphilone cluster-specific TF (AzaR), a Zn(II)2Cys6 fungus-specific TF, regulates the biosynthesis of polyketone secondary metabolites in *Aspergillus niger* and is involved in stress tolerance and conidiation pattern shift in *M. acridum* [34]. However, its role in the virulence of EPF remains unknown.

In this study, we report that MaAzaR is involved in the early stages of *M. acridum* infection. Disruption of *MaAzaR* delays cuticle penetration without affecting in vivo growth, thereby reducing its virulence. Further analysis revealed that Δ*MaAzaR* impedes cuticle penetration by delaying appressoria formation and impairing turgor pressure and hydrolytic enzyme secretion in appressoria. We also performed RNA-sequencing (RNA-seq) on WT and Δ*MaAzaR* strains after 20 h of locust hindwing growth to identify and analyze the key pathways in appressorium formation that may be regulated by MaAzaR. Our data provide important insights into host–pathogen interactions and are helpful to improve the practical application of biological pesticides.

## 2. Materials and Methods

### 2.1. Strains and Growth Conditions

The *M. acridum* strain CQMa102, utilized in this study, is preserved at the China General Microbial Culture Collection (strain No. CGMCC No. 0877). This strain was cultured at 28 °C for 15 d on 1/4 SDAY solid medium, consisting of 10 g glucose, 5 g yeast extract, 2.5 g tryptone, distilled water up to 1000 mL, and another 1.8% agar for solid medium (w/v). *L. migratoria* (Oriental migratory locust) were maintained in an insectary set at 28 °C with 60% relative humidity, following a 13 h light and 11 h dark cycle, and were fed bran and corn leaves. All media were sterilized using an autoclave at 121 °C for 30 min.

### 2.2. Bioassays

To evaluate the virulence of the three strains (WT, CP, and Δ*MaAzaR*) against *L. migratoria*, 2-day-old 5th instar locusts were subjected to topical inoculation and in vivo intra-hemocoel injection bioassays as previously described [35]. For topical inoculation, 5 μL of a conidia suspension of each strain, adjusted to a concentration of 1 × 10^7^ conidia per mL in paraffin oil, was applied to the locust dorsal plate. For the intra-hemocoel injection bioassay, the conidia suspension of each strain was prepared with 0.05% Tween 80 to a concentration of 1 × 10^6^ conidia/mL and injected into the third and fourth abdominal segments of the locusts using a microsyringe with a volume of 5 μL. Thirty insects were used for each treatment, with the experiment independently repeated three times. Insects were maintained at 28 °C with 70% humidity and fed every 12 h, and mortality was recorded. Conidiation on the host insect cadaver was assessed as previously described [36]. Briefly, locust carcasses were surface-sterilized, stored in humidified containers, and incubated at 28 °C. After spore emergence from the locust carcasses, the carcasses were photographed.

### 2.3. RNA Isolation, RT-qPCR, and RNA Sequencing

RNA extraction was performed following the manufacturer’s instructions for the Ultrapure RNA kit (CWBIO, Beijing, China). Samples were ground in liquid nitrogen and extracted with Trizol. RNA quality was assessed using a 2% agarose gel. Reverse transcription was conducted with the PrimeScript™ RT Reagent Kit (Takara, Dalian, China), and qPCR analysis was performed using SYBR^®^ Premix Ex Taq™ (Takara, Dalian, China). Relative expression levels of target genes were calculated using the 2^−ΔΔCt^ method [37], with *Actin* and *Gapdh* genes serving as reference genes for *L. migratoria* and *M. acridum*, respectively. For the transcriptome analysis, the washed (three times in distilled water) and sterilized (by autoclaving at 121 °C for 30 min) locust hind wings were placed in a centrifuge tube with 5 mL of conidium suspension (1 × 10^7^ conidia/mL) and vortexed thoroughly for at least 10 min. The wings were laid flat on a clean slide, the excess water was absorbed with filter paper, and then the wings were placed in a Petri dish lined with two layers of moistened filter paper (2 mL of sterile water was evenly added in advance). The Petri dish was incubated at 28 °C for 20 h. RNA libraries were sequenced on the Illumina Novaseq 6000 platform by LC Bio Technology Co., Ltd (Hangzhou, China). The primers used in this study are listed in Table S1.

### 2.4. Cuticle Penetration Simulation

For the simulation of cuticle penetration, wings were obtained from adult locusts, washed three times with distilled water, and sterilized by autoclaving. The sterile locust hindwings were spread flat on 1/4 SDAY plates and inoculated with 2 μL of a conidial suspension (1 × 10^7^ conidia/mL) of one of the three strains (WT, CP, or Δ*MaAzaR* mutant). The plates were incubated at 28 °C for 48 or 72 h. At each time point, the locust hindwings were removed and the plates were further incubated at 28 °C for five d. For the control groups, 2 μL of conidial suspension (1 × 10^7^ conidia/mL) of each strain was directly spotted onto 1/4 SDAY plates. Clone sizes of the strains on the plates were then compared [38].

### 2.5. Appressorium Formation and Turgor Pressure Measurement

The method for inducing appressorium formation on locust hindwings, as described in Section 2.3, was used for transcriptome sample collection. Conidia were incubated on the wings for 16, 18, 20, 24, 28, 32, or 36 h, and the rate of appressorium formation was counted in 300 conidia at each time point. Turgor pressure was estimated by adding different concentrations (0.2 to 1.2 g/mL) of PEG-8000 (polyethylene glycol) to the appressoria; after 10 min, the collapse rate of the appressoria was calculated.

### 2.6. Phenoloxidase Assay

Locusts were inoculated as previously described in Methods Section 2.4. Hemocoel was extracted from the locusts (10 μL per locust, five insects per treatment) at the appropriate times, with a total of 50 μL of hemocoel being prepared. After centrifugation at 30× *g* for 10 min, the supernatant (hemolymph) was aspirated from the pellet and placed on ice. Subsequently, 300 μL of 0.1 M potassium dihydrogen phosphate buffer (pH 6.0, 28 °C), 10 μL of 0.01 M levodopa, and 10 μL of hemolymph were added to individual wells of a 96-well plate, which was then shaken to ensure uniform mixing of the solution. One unit of PO enzyme activity is defined as an increase of one OD_485_ unit per mg of protein per min. Three biological replicates were performed for each sample [35].

### 2.7. *In Vitro* Culture of Hyphal Bodies and its DNA Content Quantification

Conidia from WT were inoculated in 1/4 SDAY liquid medium and incubated in a shaker at 28 °C for 3 d. Mycelium was ground in liquid nitrogen after filtration, and the genome of the mycelium was extracted following the instructions on the OMEGA Genome Extraction Kit (Omega, GA, USA). A gradient dilution (10–100 pg) of the genome of WT was then used as a template for real-time quantitative PCR (qPCR) using *M. acridum*-specific ITS primers to make a standard curve.

Hemolymph was extracted from healthy fifth instar *L. migratoria* (10 µL per insect, 50 insects per group, a total of 500 µL hemolymph). Centrifugation at 600 rpm for 10 min was performed to separate the hemocytes from the hemolymph. A total of 2 μL of conidia suspension (1 × 10^6^) of each strain was added to the supernatant. The mixture was then incubated at 28 °C with shaking. HBs DNA was extracted 1 to 4 days post-inoculation (dpi) according to the instructions of the OMEGA Genome Extraction Kit (Omega, GA, USA) and was used as a template for the detection of HBs Ct values using *M. acridum*-specific ITS primers, which were then substituted into a standard curve to derive the genomic concentration of HBs. There were three replicates per group, and the experiment was repeated three times independently [39].

### 2.8. Data Analysis

All experiments were carried out with three independent replicates. Statistical analysis was performed using SPSS (SPSS Inc., Chicago, IL, USA, version 26.0 for Windows). Data were expressed as mean ± standard error of the mean (SEM). One-way ANOVA was used to compare more than two samples, and the Tukey test was conducted as the multiple pairwise comparison test once ANOVA was shown to be significant.

## 3. Results

### 3.1. Disruption of MaAzaR Reduces the Virulence of M. acridum by Impeding Cuticle Penetration

**Figure 1:**
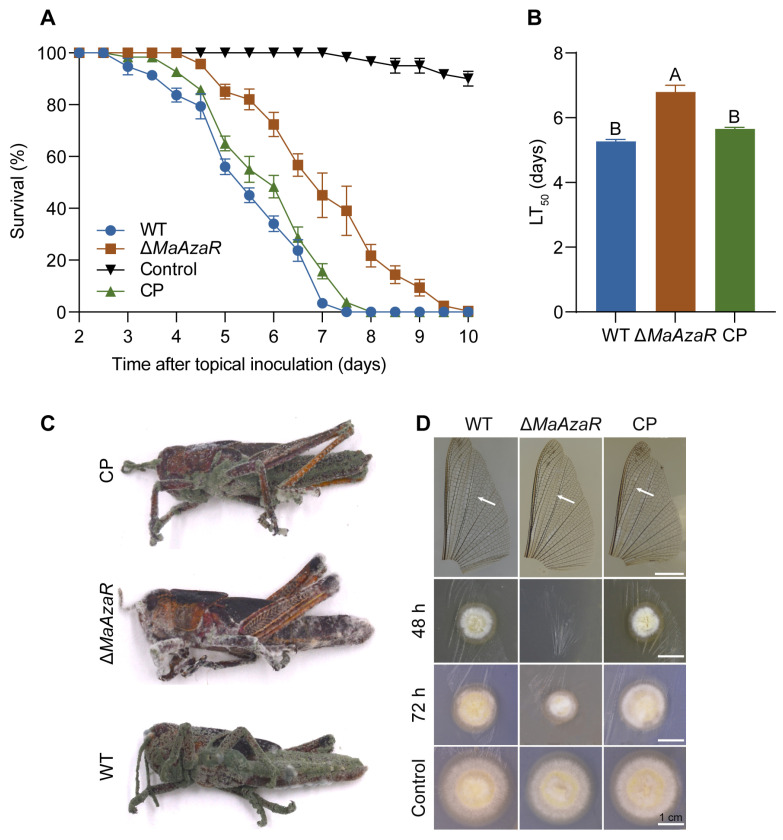
Insect bioassay and simulation of *Metarhizium acridum* cuticle penetration. (**A**) Survival of *Locusta migratoria manilensis* topically inoculated with each strain. (**B**) Calculated LT_50_ (median lethal time, i.e., time until death) values of *L. migratoria* in the topical inoculation bioassay. (**C**) Image of locust cadaver 7 days post-death in topical inoculation bioassay. (**D**) Cuticle penetration simulation assay; the white arrow points to the location of the conidia vaccinate. Data points represent the mean ± standard error of the mean (SEM). The same capital letters above two bars indicate no significant difference between the samples by one-way ANOVA and Tukey test (*p* > 0.01).

To investigate the function of MaAzaR in *M. acridum*, the *MaAzaR* knockout (Δ*MaAzaR*) and complementation (CP) strains were constructed in previous research [34]. To ask whether MaAzaR affects *M. acridum* virulence, we used *L. migratoria* as the host to perform a topical inoculation bioassay to simulate natural infection. The results revealed a significantly reduced mortality rate in locusts inoculated with Δ*MaAzaR* compared to the WT and CP strains. At 8 dpi, all locusts in the other treatments had succumbed, while 20% of locusts treated with Δ*MaAzaR* remained alive, with complete mortality occurring only at 10 dpi. Further analysis of median lethal time (LT_50_) showed an average increase of 1.5 d in LT_50_ for the Δ*MaAzaR* knockout strain compared to the WT (Figure 1A,B). Visual inspection of locust cadavers killed by the WT and CP strains exhibited robust conidiation 7 days post-death (Figure 1C), disruption of *MaAzaR* further impairing *M. acridum* prevalence in the locust population.

The insect integument is the first obstacle against fungal invasion [21]. Cuticle penetration is the initial step for the successful infection of the host by the EPF. Therefore, we simulated the process of *M. acridum* infecting locusts using locust hind wings, as described in Methods 2.4. At 48 h post-inoculation (hpi), both the WT and CP strains successfully penetrated the hind wings of locusts. However, the knockout strain could only achieve penetration at 72 hpi. This suggests that MaAzaR may influence the virulence of *M. acridum* by affecting the process of host cuticle penetration (Figure 1D).

### 3.2. MaAzaR Influences Cuticle Penetration Process by Affecting Appressoria

**Figure 2:**
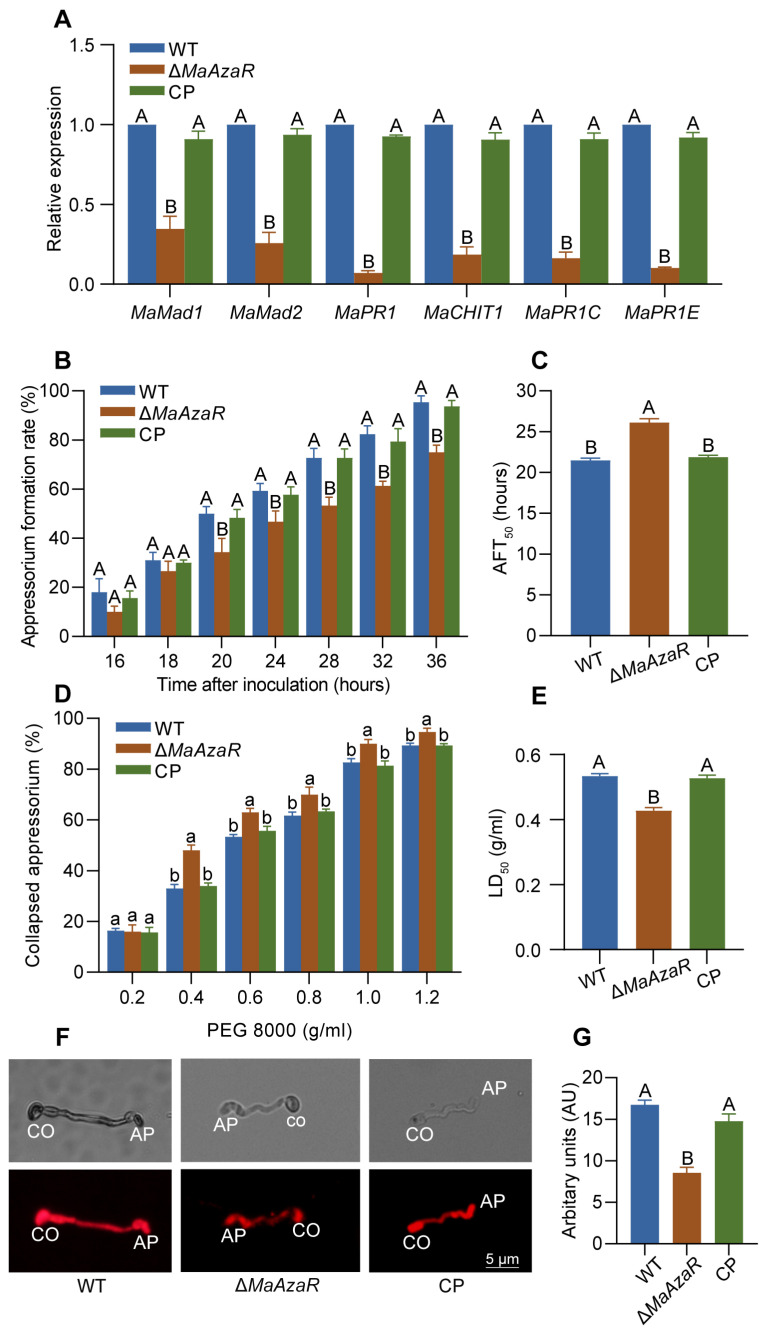
The deletion of *MaAzaR* affects cuticle penetration by influencing appressoria. (**A**) Relative expression of adhesin-like proteins and extracellular hydrolytic enzymes in appressoria after 24 h post-inoculation (hpi) with locust’s hind wings. (**B**) Appressoria formation rate on locust wings from 16 to 36 hpi of each strain. (**C**) The time required for 50% appressoria formation (AFT_50_). (**D**) The collapsed rate of appressoria treated with different concentrations of PEG-8000. (**E**) Concentration of PEG-8000 to make 50% of appressorium collapse (LD_50_). (**F**) Nile Red staining of lipid droplets in the appressorium (AP) and conidium (CO). (**G**) Fluorescence of lipid droplet staining with Nile Red was quantified. Data points represent the mean ± standard error of the mean (SEM). The same lowercase letters and capital letters above two bars indicate no significant difference between the samples by one-way ANOVA and Tukey test (*p* > 0.05 and *p* > 0.01, respectively).

We firstly quantified the expression levels of adhesion-related genes, including *Mad1* and *Mad2*, and observed that the expression levels of these genes in Δ*MaAzaR* were significantly lower than those in the WT and CP strains, with reductions of at least 50% (Figure 2A). Differentiation of appressoria is a prerequisite for infection by most EPF [22]. So, we then quantified the appressoria formation rates of different strains and found that Δ*MaAzaR* formed less appressoria than WT (Figure 2B). The AFT_50_ (the time required for a 50% appressoria formation rate) for Δ*MaAzaR* was 4.6 h longer than that for WT (Figure 2C). Additionally, the turgor pressure of the appressoria formed by Δ*MaAzaR* strain was lower than that of the WT strain (Figure 2D,E). Then, we stained appressoria with Nile Red and observed that the appressoria formed by Δ*MaAzaR* had fewer lipid droplets compared to those formed by WT (Figure 2F,G). Also, the relative expression of proteases *MaPR1*, *MaPR1C*, and *MaPR1E* and the chitinase *MaCHIT1* in Δ*MaAzaR* during appressoria formation is also significantly lower than those in the WT and CP strains (Figure 2A). These results suggest that the absence of *MaAzaR* leads to slowing down of the cuticle penetration process by reducing the formation of appressoria as well as impairing turgor pressure and hydrolytic enzymes of appressoria.

### 3.3. Disruption of MaAzaR Induces Higher Host Immune Responses During Cuticle Penetration

**Figure 3:**
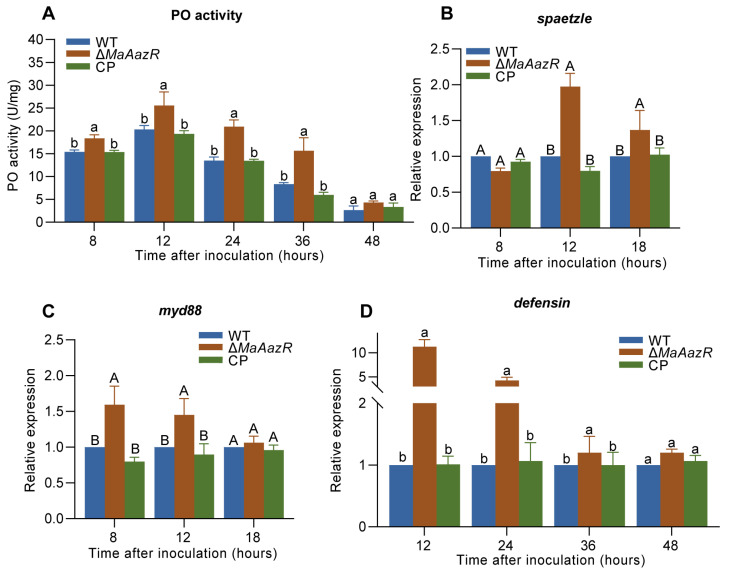
Disruption of *MaAzaR* induces higher host immune responses during cuticle penetration. (**A**) Phenoloxidases (PO) enzyme activity of locust hemolymph after topical inoculation with each strain. (**B**) Relative expression of *spaetzle* at 8, 12, and 18 h post-inoculation (hpi) after topical inoculation. (**C**) Relative expression of *myd88* at 8, 12, and 18 hpi after topical inoculation. (**D**) Relative expression of *defensin* at 12, 24, 36, and 48 hpi after topical inoculation. Data points represent the mean ± standard error of the mean (SEM). The same lowercase letters and capital letters above two bars indicate no significant difference between the samples by one-way ANOVA and Tukey test (*p* > 0.05 and *p* > 0.01, respectively).

During the process of EPF infection, the host protects itself from fungal development through the collaboration between cellular immunity and humoral immunity [21,22]. We assessed the host’s immune response by measuring the PO enzyme activity, as well as the expression levels of Toll pathway genes, *spaetzle* and *myd88*, and antimicrobial peptide, *defensin*. The results showed that the PO activity in locusts inoculated with the Δ*MaAzaR* strain was significantly higher than that in locusts inoculated with the WT and CP strains from 8 hpi to 36 hpi (Figure 3A). Additionally, the relative expression levels of Toll pathway genes and the antimicrobial peptide *defensin* induced by Δ*MaAzaR* were higher compared to those induced by WT (Figure 3B–D). Those suggest that the absence of *MaAzaR* may induce a stronger host immune response during the process of cuticle penetration.

### 3.4. The Absence of MaAzaR Does Not Impair In Vivo Growth Stage of M. acridum

**Figure 4:**
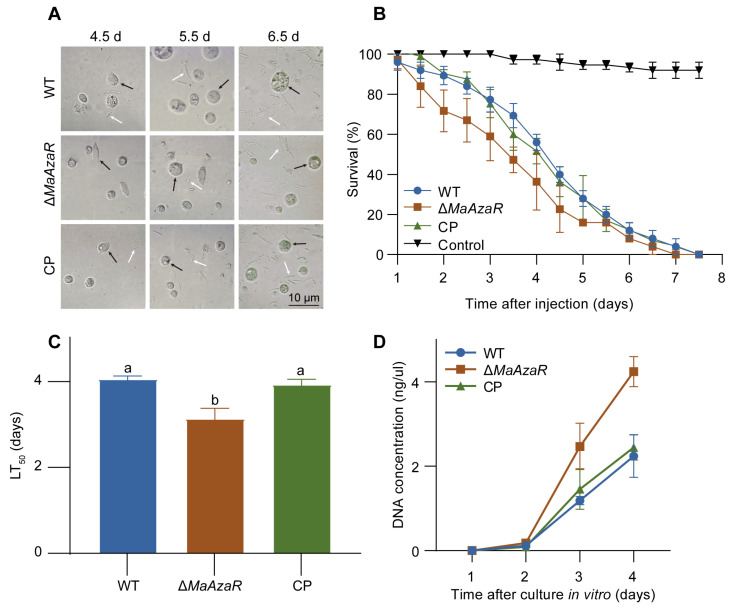
In vivo growth stage does not cause the decreased virulence of Δ*MaAzaR* strain. (**A**) Microscopic images of hyphal bodies and insect hemocytes in hemolymph after topical inoculation. Black arrow: insect hemocytes. White arrow: hyphal bodies. (**B**) Survival of *Locusta migratoria manilensis* following intrahemocoel injection with different strains. (**C**) Calculated LT_50_ (median lethal time) values of *L. migratoria* in the intrahemocoel injection bioassay. (**D**) Quantification of DNA concentration of in vitro cultured hyphal bodies. Data points represent the mean ± standard error of the mean (SEM). The same lowercase letters above two bars indicate no significant difference between the samples by one-way ANOVA and Tukey test (*p* > 0.05).

Upon successful penetration of the host insect’s cuticle and entry into the hemocoel, fungal hyphae converted to yeast-like HBs to adapt to the host hemocoel. Hemolymph samples were collected during the topical inoculation bioassay to observe HBs. At 4.5 dpi, HBs were observed in the hemolymph of locusts treated with the WT and CP strains. However, in locusts treated with the Δ*MaAzaR* strain, HBs were observed at 5.5 dpi, approximately 1 d later than those treated with WT. Furthermore, the number of HBs observed in locusts inoculated with Δ*MaAzaR* was lower compared to those formed by the WT strain (Figure 4A). The reduction in HBs, is it due to an extended duration of penetration, or is it attributed to growth inhibition within the hemolymph? We directly injected conidia into the hemocoel of locusts, bypassing the process of cuticle penetration, to simulate the growth of different fungal strains within the hemolymph. The results showed that the absence of *MaAzaR* resulted in accelerated locust mortality (Figure 4B,C). This revealed that the absence of *MaAzaR* does not impair the in vivo growth capability, and it even led to an enhancement of growth within the hemolymph. Next, we collected the healthy locust hemolymph for conidia cultivation in vitro, simulating the growth conditions of different fungal strains within the hemolymph. We then quantified the growth ability of different strains by measuring fungal DNA concentrations in the locust hemolymph. Similar results were observed (Figure 4D).

### 3.5. Transcriptomic Insights into the Roles of MaAzaR in Appressorium Formation

**Figure 5:**
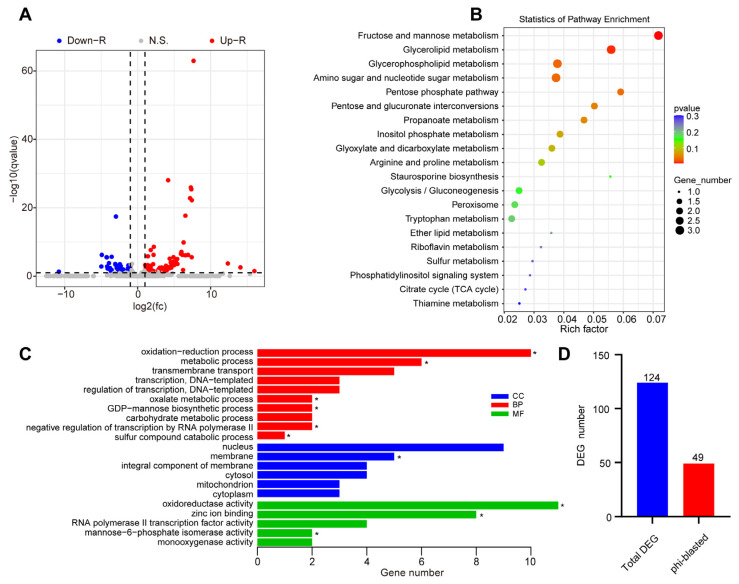
Transcriptomic analysis of MaAzaR in *Metarhizium acridum*. (**A**) Volcano plot of differentially expressed genes (DEGs) between the knockout mutant Δ*MaAzaR* and the wild-type (WT) strain grown 20 h in locust hindwings. (**B**) Enrichment of total DEGs to Kyoto Encyclopedia of Genes and Genomes (KEGG) pathways. (**C**) Enrichment of total DEGs to gene ontology (GO) item. BP, biological process; CC, cellular component; MF, molecular function. * indicates the significantly enriched terms (*q* < 0.05). (**D**) Quantification of total DEGs and homolog genes identified in the Pathogen–Host Interactions (PHI) database (phi-blasted DEGs).

As mentioned above, the absence of *MaAzaR* resulted in a reduction in the virulence of *M. acridum* by decreasing the turgor pressure, relative expression of hydrolytic enzymes, and formation rate of appressoria. Transcriptomic analysis was performed to reveal differentially expressed genes (DEGs) in Δ*MaAzaR* and WT strains grown on wings for 20 hpi. There were 124 DEGs in Δ*MaAzaR* compared to WT (|log2FC| > 1, where FC = fold-change, *q* < 0.05): 87 upregulated and 37 downregulated (Figure 5A, Table S2). The Kyoto Encyclopedia of Genes and Genomes (KEGG) pathways of total DEGs were mainly enriched in various metabolic pathways, including fructose and mannose metabolism, glycerolipid metabolism, glycerophospholipid metabolism, and amino sugar and nucleotide sugar metabolism (Figure 5B). Gene ontology (GO) annotation was performed to reveal the function of DEGs. The results showed significant enrichment of GO terms such as oxidation–reduction process, metabolic process, membrane, oxidoreductase activity, and zinc ion binding (Figure 5C). To mine important genes associated with the pathogenicity of fungal infestations affected by MaAzaR, the DEGs were subjected to BLAST alignment in the Pathogen–Host Interactions (PHI) database (http://www.phi-base.org/index.jsp), which contains the reported pathogenicity, virulence, and effector genes of pathogenic microorganisms. A total of 49 DEGs (E-value: 1 × 10^−5^) were successfully matched to homologs in the database, implying that approximately 40% of the total DEGs were associated with virulence (Figure 5D, Table S3). This suggests that MaAzaR may be a crucial transcription factor associated with virulence by regulating metabolite synthesis.

## 4. Discussion

Zn(II)2Cys6 transcription factors are unique to the fungal kingdom and have diverse functions. It has contributed to virulence in various pathogenic fungi. VpFSTF1, a fungal-specific TF with a Zn(II)2Cys6 binuclear cluster, is essential for fruiting body formation and virulence in *Valsa pyri* [40]. Zcf15 and Zcf29 are required for the virulence of human pathogen *Candida albicans* [41]. In our study, deleting *MaAzaR* decreased its virulence to *L. migratoria* under natural infection and resulted in fewer conidia on cadavers, further influencing its prevalence in the locust population.

The insect integument serves as the primary barrier against fungal invasion [21], and cuticle penetration is the initial step essential for successful host infection by the pathogenic fungus. Our results indicate that the absence of *MaAzaR* impairs *M. acridum*’s ability to penetrate the cuticle. The appressorium is the main infective structure of a pathogenic fungus, facilitating invasion of the host from the cuticle [21]. Previous research has shown that disruption of *GPF1*, *PCF1*, *CNF1*, and *CCA1* affects appressorium formation and is required for virulence in *M. oryzae* [42]. In *Metarhizium*, the absence of *Mamk1*, *Makatg1*, and *Mrark1* decreased the rate of appressorium formation and thus reduced the virulence of knockout strains [43,44,45]. In our research, the absence of *MaAzaR* delayed appressorium formation. Appressoria establish adequate turgor pressure through the transport of lipids to the developing appressoria, which are subsequently degraded to glycerol. This process increases turgor pressure, serving as the driving force for mechanical penetration [22]. This mechanism enables the upward growth of penetration pegs and facilitates the breach of the host cuticle [21]. During cuticle penetration, appressoria produce a variety of extracellular hydrolytic enzymes, including lipases, proteases, and chitinases, to facilitate penetration [21,22]. We found that the loss of *MaAzaR* reduced lipid droplet content and turgor pressure in appressoria. The relative expression of the extracellular hydrolase gene in Δ*MaAzaR* was also reduced compared to the WT strain. Those results suggest that MaAzaR is an important transcription factor during appressoria formation.

Transcriptome analysis of appressorium formation revealed that the KEGG pathways of the DEGs primarily highlighted various metabolic pathways, including fructose and mannose metabolism, glycerolipid metabolism, glycerophospholipid metabolism, and amino sugar and nucleotide sugar metabolism. DEGs involved in fructose and mannose metabolism, such as MAC_03237, MAC_05390, and MAC_07720, are associated with the integrity of the fungal cell wall or polysaccharide capsule, transportation, and catalysis, influencing nutrient absorption, morphological growth, and virulence [46,47,48,49]. Approximately 40% of the total DEGs have homolog genes in the PHI database. Among these, MAC_06847 (log2FC = −10.8) encodes a serine protease, its homologous gene, *Bcser2,* contributes to hyphal growth, sclerotial formation, and conidiation in *Botrytis cinerea*. When *Bcser2* is disrupted, *B. cinerea* loses the ability to infect intact plant leaves of *Arabidopsis* and *Solanum lycopersicum* L. [50]. MAC_05902 (log2FC = −4.11) has a homologous gene in the PHI database, *Ss-oas1*, which encodes an oxaloacetate acetylhydrolase. The Δ*Ss-oah1* mutant of *Sclerotinia sclerotiorum* cannot infect efficiently due to a defect in appressorium development [51]. This reveals the crucial role of MaAzaR in regulating appressorium formation, but the specific pathways it regulates require further research. A previous study found that MaAzaR plays a role in conidiation pattern shifts and stress tolerance in *M. acridum* [34]. Transcriptomic data from conidia production and appressoria formation revealed three identical DEGs: MAC_00247, MAC_01894, MAC_08357. These DEGs are likely downstream targets of MaAzaR, although further investigation is needed to confirm their direct regulation.

The host protects itself against fungal development through cellular immunity and humoral immunity [21,22]. During cuticle penetration, the host activates the prophenoloxidase (PPO) system and antimicrobial compounds in the cuticle to inhibit fungal development. PO directly attaches to and melanizes the fungal cell wall to impede hyphal growth [25]. Following conidial attachment to locusts, increased expression of Toll pathway genes, such as *Spatzle*, *Toll9*, and *myd88*, is observed in the fat bodies during *M. acridum* germination [52]. We observed increased immune responses, including higher PO activity and increased expression levels of *Spatzle*, *myd88*, and *defensin,* induced by Δ*MaAzaR* during cuticle penetration. During the in vivo growth stage, fungal hyphae convert to yeast-like hyphal bodies to adapt to the host hemocoel [21,22]. The fungal cell wall remodels to evade the host immune response [21,52]. Furthermore, hyphal bodies release toxic substances while consuming host nutrients, ultimately leading to host death [21]. During this phase, the absence of *MaAzaR* does not impair the growth of *M. acridum* in the hemolymph of locusts, and it even enhances growth within the hemolymph.

## 5. Conclusions

MaAzaR impacts the virulence of *M. acridum* by affecting the cuticular penetration process rather than the intracellular growth within the host. During cuticle penetration, disruption of *MaAzaR* slows the formation rate of appressoria and impairs their turgor pressure and the expression of hydrolytic enzymes. The present study provides an important theoretical basis for selecting EPF strains and for promoting and utilizing EPF.

## Data Availability

Clean data were deposited in the NCBI Sequence Read Archive database (accession number: PRJNA883628).

## Supplementary Materials

The following supporting information can be downloaded at: https://www.mdpi.com/article/10.3390/jof10080564/s1, Table S1. Primers used in the study. Table S2. The DEGs in appressoria formation of Δ*MaAzaR* compared to WT. Table S3. DEGs which identified homolog genes in the Pathogen–Host Interactions (PHI) Database.
